# Adapting UK Biobank imaging for use in a routine memory clinic setting: The Oxford Brain Health Clinic

**DOI:** 10.1016/j.nicl.2022.103273

**Published:** 2022-11-21

**Authors:** Ludovica Griffanti, Grace Gillis, M. Clare O'Donoghue, Jasmine Blane, Pieter M. Pretorius, Robert Mitchell, Nicola Aikin, Karen Lindsay, Jon Campbell, Juliet Semple, Fidel Alfaro-Almagro, Stephen M. Smith, Karla L. Miller, Lola Martos, Vanessa Raymont, Clare E. Mackay

**Affiliations:** a Department of Psychiatry, University of Oxford, United Kingdom; b Oxford Health NHS Foundation Trust, Oxford, United Kingdom; c Nuffield Department of Clinical Neurosciences, University of Oxford, United Kingdom; d Wellcome Centre for Integrative Neuroimaging, University of Oxford, United Kingdom; e Oxford University Hospitals NHS Trust, Oxford, United Kingdom

**Keywords:** UK Biobank, structural MRI, Magnetic resonance imaging, Memory clinic, Dementia

## Abstract

The Oxford Brain Health Clinic (BHC) is a joint clinical-research service that provides memory clinic patients and clinicians access to high-quality assessments not routinely available, including brain MRI aligned with the UK Biobank imaging study (UKB).

In this work we present how we 1) adapted the UKB MRI acquisition protocol to be suitable for memory clinic patients, 2) modified the imaging analysis pipeline to extract measures that are in line with radiology reports and 3) explored the alignment of measures from BHC patients to the largest brain MRI study in the world (ultimately 100,000 participants).

Adaptations of the UKB acquisition protocol for BHC patients include dividing the scan into core and optional sequences (i.e., additional imaging modalities) to improve patients’ tolerance for the MRI assessment. We adapted the UKB structural MRI analysis pipeline to take into account the characteristics of a memory clinic population (e.g., high amount of white matter hyperintensities and hippocampal atrophy). We then compared the imaging derived phenotypes (IDPs) extracted from the structural scans to visual ratings from radiology reports, non-imaging factors (age, cognition) and to reference distributions derived from UKB data.

Of the first 108 BHC attendees (August 2020-November 2021), 92.5 % completed the clinical scans, 88.0 % consented to use of data for research, and 43.5 % completed the additional research sequences, demonstrating that the protocol is well tolerated. The high rates of consent to research makes this a valuable real-world quality research dataset routinely captured in a clinical service. Modified tissue-type segmentation with lesion masking greatly improved grey matter volume estimation. CSF-masking marginally improved hippocampal segmentation. The IDPs were in line with radiology reports and showed significant associations with age and cognitive performance, in line with the literature. Due to the age difference between memory clinic patients of the BHC (age range 65–101 years, average 78.3 years) and UKB participants (44–82 years, average 64 years), additional scans on elderly healthy controls are needed to improve reference distributions. Current and future work aims to integrate automated quantitative measures in the radiology reports and evaluate their clinical utility.

## Highlights

• We adapted UK Biobank brain MRI imaging for routine use in memory clinic.
• The acquisition protocol is well tolerated and provides high-quality data.
• The modified analysis pipeline improves grey matter and hippocampus segmentation.
• Volumes are aligned with radiology reports and associated with age and cognition.
• Data from older healthy controls are needed to improve reference distributions.

## 1 Introduction

Brain magnetic resonance imaging (MRI) plays a key role in the diagnosis and evaluation of patients suspected of having dementia (Filippi et al., 2012, Staffaroni et al., 2017, Vernooij et al., 2019). Atrophy, specifically medial temporal lobe atrophy, has been included in the diagnostic guidelines for Alzheimer’s since 2011 (McKhann et al., 2011), and distinct atrophy patterns can point towards a specific underlying diagnosis of dementia subtype (Frisoni et al., 2010, McKhann et al., 2011, Staffaroni et al., 2017). More recently, there has been increasing awareness of the importance of vascular pathology as another key threat to cognitive health (Dickie et al., 2018, Gorelick et al., 2011, Smith et al., 2019).

Despite our understanding of structural changes in dementia, neuroimaging modalities are under-exploited in the clinical setting. In the UK, most people with memory problems (above 65 years old and with normal presentation) are referred to psychiatry-based memory clinics. These services typically do not have access to the same assessments (MRI and neuropsychology) as more specialist neurology-based clinics. Rather than to diagnose dementia, CT is most commonly used to exclude other pathologies, which account for a small proportion of cases (Scheltens et al., 2002, van Straaten et al., 2004). Specific atrophy patterns and the presence of vascular pathology are better assessed with MRI compared with CT (Scheltens et al., 2002, van Straaten et al., 2004).

The results of brain scans are usually reported by a radiologist without the benefit of a standardised dementia-specific structure, leading to highly variable content across patients. Consortia and working groups are making an effort to standardise the structure of radiology reports, include information about vascular pathology, and often suggest the use of semi-quantitative scales (Filippi et al., 2012, Smith et al., 2019, Vernooij et al., 2019). Including quantitative measures extracted from MRI in radiology reports has the potential to increase the accuracy of dementia diagnosis and prognosis (Bosco et al., 2017, Vernooij et al., 2018), reduce inter-rater variability, and improve workflows (Goodkin et al., 2019). However, despite being widely used in dementia research, MRI and quantitative measures derived from MRI are not commonly used in routine clinical assessments.

In research settings, various neuroimaging software suites have been developed and widely implemented to derive quantitative measures from brain MRI (e.g. cortical and hippocampal volume, volume of white matter hyperintensities – WMHs). More recently, with imaging now being more regularly included in large population cohorts, there are efforts to standardise these measurements and make them interpretable by non-imaging experts to maximise the usability of brain health information. A good example is the UK Biobank (UKB) study, which will ultimately include multi-organ (including brain) imaging for 100,000 participants. This can be combined with lifestyle, health and genetic data to produce predictive models for late life brain health (Littlejohns et al., 2020, Miller et al., 2016). The brain MRI UKB image processing pipeline (Alfaro-Almagro et al., 2018) automatically extracts thousands of measurements, so-called imaging derived phenotypes (IDPs). The pipeline uses tools largely from the FSL and FreeSurfer software libraries, which are used by thousands of research laboratories world-wide. However, these tools have primarily been validated only in a research context. The applicability of these tools and the UKB pipeline to an unselected clinical population remains unclear.

The Oxford Brain Health Clinic (BHC)(O’Donoghue et al., 2022a) aims to address this disconnect between the research and clinical domains, whilst providing an ideal clinical setting to validate MRI analysis tools. A joint clinical-research service that opened in August 2020, the BHC augments current NHS psychiatry-led memory clinic services to give patients and clinicians access to high-quality assessments not routinely available, including MRI instead of CT. MRI sequences used in the BHC are identical to, or compatible with, the UK Biobank imaging study (Miller et al., 2016), enabling us to explore the alignment of BHC results with the larger UKB imaging dataset.

Our ambition to incorporate high quality MRI acquisition and analysis is shared by a number of centres of excellence, research studies, and by a growing number of clinical research organisations and spinouts. Notable examples are the Quantitative Neuroradiology initiative (Goodkin et al., 2019) and the work by Biondo and colleagues (Biondo et al., 2022), using routine T1-weighted images from memory clinic patients in South London and Maudsley. A number of multi-site initiatives are also in progress (e.g. the Cambridge-led Quantitative MRI in NHS Memory Clinics study). The potential commercial opportunity is being exploited by a growing number of companies (e.g. Icometrix, Brainminer, Oxford Brain Diagnostics, Cortechs.ai, AINOSTICS, just to name a few). The specific contribution of the Oxford BHC relates to the population (non-selected clinical referrals from a psychiatry-led service), the research consenting model (patient choice with a very high consent rate) and the adaptation of the UK Biobank MRI acquisition and analysis methodology, which directly aligns this late-life clinical population with the largest epidemiological imaging study in the world. The population and consenting model are described elsewhere (O’Donoghue et al., 2022a), while this paper focuses on the methodological challenges of aligning brain MRI data between BHC and UKB. In particular, we sought to address three questions: 1) is the UKB acquisition protocol well tolerated by patients? 2) is the UKB pipeline robust and generalisable to a real-life memory clinic population? 3) can UKB data be used as a reference population for memory clinic patients?

In this work we describe how we have adapted the UKB protocol for use in the BHC and evaluated the tolerability of the BHC protocol for memory clinic patients both in terms of compliance and image quality. We assess the performance of the UKB analysis pipeline and optimise the necessary automated segmentation tools for use with this patient group. Finally, we compare the characteristics of BHC patients with UKB participants and discuss challenges and opportunities of using UKB as normative distributions and implementing quantitative measures in BHC reports.

## 2 Methods

### 2.1 Patient population

Patients from Oxfordshire who have been referred to Oxford Health NHS Foundation Trust Memory Clinics are triaged by the duty psychiatrist (RM) for referral to the Brain Health Clinic for assessments prior to their memory clinic appointment. Selection at the triage stage is based on clinical need and MRI safety screening. The GP referral and notes are examined for evidence of MRI safety contraindications and the duty psychiatrist speaks to the patient by phone to assess suitability for scanning. There is no explicit age cut-off for a BHC referral, but a clinical decision is made about the benefit of a BHC visit for patients who are deemed too frail for advanced scanning or where dementia is well established.

At the BHC, patients undergo extensive brain health assessments, including cognitive assessments, physiological measurements, questionnaires, and a 3 T MRI scan.

Patients are also offered three ways to take part in research: 1) to share their clinical data for research use, 2) to undergo additional assessments during their visit and 3) to be informed about future opportunities to participate in studies and trials. All research is optional, and patients that choose not to take part in research still complete the NHS assessment at the BHC. The BHC Research Database was reviewed and approved by the South Central – Oxford C research ethics committee (SC/19/0404).

For more details on the non-imaging assessments performed and the research consent process, please refer to (O’Donoghue et al., 2022a).

### 2.2 BHC MRI protocol

The UKB brain MRI protocol was optimised to produce high-quality images in as short a time as possible, to accommodate the large N (Miller et al., 2016, Smith, 2020), making it an ideal candidate as a clinical protocol.

The BHC brain MRI protocol was designed to match as closely as possible the UKB, with some adaptations intended to make the scan more tolerable for memory clinic patients and to prioritise the collection of images that are currently most useful to aid dementia diagnosis. Table 1 illustrates the BHC MRI protocol compared with the UKB protocol. The full protocol details are openly available on the WIN MR Protocols Database (O’Donoghue et al., 2022b).

**Table 1:** BHC MRI protocol: acquisition details and comparison with UKB protocol.

Modality	Acquisition time	Resolution	Matrix	Key Parameters	UKB Match
BHC core clinical protocol	16:29				
Localiser	0:14				No
Diffusion MRI (dMRI 3-scan trace)	0:43	0.8x0.8x4 mm	260x260x27	TR = 3800 ms, 3 dirs, b = 0, 1000 s/mm^2^	N/A
Susceptibility-weighted MRI (swMRI)	4:46	0.8x0.8x1.5 mm	256x288x80	TE1/TE2/TR = 10/20/30 ms, R = 2	Adapted
T1 (MPRAGE)	4:54	1.0x1.0x1.0 mm	256x256x208	TI/TR = 800/2000 ms, R = 2	Exact
T2-FLAIR (SPACE)	5:52	1.0x1.0x1.05 mm	256x256x192	TI/TR = 1800/5000 ms, R = 2	Exact

BHC research protocol	21:17				
ASL localiser (time of flight)	0:42				N/A
Diffusion MRI (dMRI)	7:08	2.0x2.0x2.0 mm	104x104x72	TR = 3600 ms, 50 dirs/shell, b = 0,1000,2000 s/mm^2^, MB = 3, blip-reversed b = 0	Exact
Arterial spin labelling (PCASL)	7:17	3.4x3.4x4.5 mm	64x64x24	TE/TR = 14/4400 ms, tag = 1400 ms, seven PLDs = 250,500…1750 ms, 1 M0 calibration image	Not in UKB initial protocol
Resting state fMRI (rfMRI)	6:10	2.4x2.4x2.4 mm	88x88x64	TE/TR = 39/735 ms, α = 52°, MB = 8	Exact

MPRAGE = Magnetization Prepared RApid Gradient Echo; FLAIR = Fluid-attenuated inversion recovery; SPACE = Sampling Perfection with Application optimized Contrasts using different flip angle Evolution; ASL = Arterial Spin Labeling; PCASL = pseudo-continuous ASL; TR = Repetition time; TE = Echo time; TI = Inversion time; R = In-plane acceleration factor; MB = Multi-band acceleration factor; α = flip angle; PLD = postlabeling delay; M0 = equilibrium magnetization, required for ASL quantification. Full MRI protocols are available online (BHC: https://open.win.ox.ac.uk/protocols/stable/6974395a-3745–4861-b8cc-1887e787d1c4 (O’Donoghue et al., 2022b); UKB: https://open.win.ox.ac.uk/protocols/stable/d2b297c3-4a4f-4fde-9b7c-7a8ae6e5fa83).

The scanner used in UKB is a Siemens Skyra 3 T running software platform VD13A, with a 32-channel RF receive head coil. The BHC protocol was setup on a Siemens Prisma 3 T running VE11C, with a 32-channel head coil. The Prisma is a slightly newer, higher-specification scanner than the Skyra and is running a newer software platform, hence is able to run the UKB sequences and protocol.

#### 2.2.1 Subdivision into clinical and research sequences

Of the MRI modalities included in the UKB protocol, some are routinely used in clinical settings, while others are currently used mainly for research purposes.

Leveraging the joint clinical-research nature of the BHC we divided the UKB protocol into two sets of sequences: a core clinical protocol including the sequences that are compatible with current radiological examinations of memory clinic patients, and a research protocol that patients can opt-in to receive if they consent to additional research assessments.

#### 2.2.2 BHC core clinical protocol

The core clinical protocol has a total acquisition time of 16:29 mins and includes a T1-weighted scan, a T2-weighted Fluid Attenuated Inversion Recovery (FLAIR) scan, a diffusion-weighted scan (dMRI) and a susceptibility-weighted (swMRI) scan.

The T1 and T2-FLAIR scans are exactly matched with UKB protocol. These high-resolution structural scans (1 mm isotropic) allow clear depiction of brain anatomy, with high contrast between grey and white matter (T1) and highlight alterations to tissue compartments typically associated with pathology (T2-FLAIR). Neither the BHC nor the UKB protocol include a T2-weighted (non-FLAIR) scan. This decision was a compromise between scanning time and clinical utility. T2-FLAIR and T2-weighted are very similar sequences, both in contrast and scan duration. T2-FLAIR offers better contrast than a T2-weighted scan for white matter pathology and infarcts, which are very important in the context of dementia diagnosis. The utility of T2-weighted scans would be more for non-neurodegenerative pathology and infratentorial or thalamic infarcts. In order to maximise the amount of clinically relevant information in a short scan, the preference was to include a T2-FLAIR and not a T2-weighted scan.

We then acquire a short dMRI scan (43 s) with just 3 orthogonal diffusion directions, as commonly done in clinical practice to evaluate mean diffusivity and detect areas of restricted diffusion for the assessment of ischaemic injury or prion disease.

The susceptibility-weighted sequence was modified from the UKB protocol to obtain higher resolution images (1.5 mm slice thickness vs 3 mm in UKB protocol), useful for the assessment of venous vasculature, microbleeds or aspects of microstructure (e.g., iron, calcium and myelin).

#### 2.2.3 BHC research protocol

The research protocol includes the UKB dMRI and resting state functional MRI (rfMRI) sequences, and an arterial spin labelling (ASL) sequence. The acquisition time for these optional research sequences is 21:17 mins.

The UKB dMRI sequence includes 100 diffusion-encoding directions across 2b-shells (50x b = 1000 s/mm^2^, 50x b = 2000 s/mm^2^) with multiband acceleration factor of 3. With respect to the clinical 3-scan dMRI, this sequence enables finer measurement of the random motion of water molecules to infer information about white matter (WM) microstructural properties and delineate the gross axonal organisation of the brain.

The UKB rfMRI sequence includes 490 timepoints (volumes) acquired with 2.4-mm isotropic spatial resolution and TR = 0.735 s, with multiband acceleration factor of 8. Resting-state functional MRI measures changes in blood oxygenation associated with intrinsic brain activity (i.e., in the absence of an explicit task or sensory stimulus). During resting-state scans, subjects are instructed to look at a crosshair, blink normally and try not to fall asleep.

We did not include the task fMRI sequence from UKB as it was not designed for this type of population. Instead, given the impact of vascular risk factors and vascular pathology on dementia (de la Torre, 2012, Iturria-Medina et al., 2016), it was deemed particularly important to look at vascular brain health in the BHC. We therefore included an Arterial Spin Labelling (ASL) sequence to look at brain perfusion, preceded by a time of flight (TOF) acquisition to localise neck vessels. We used a 2D-multi-slice EPI readout, with pseudo-continuous ASL tag duration of 1400 ms, seven post labelling delays (PLDs = 250,500…1750 ms), seven label/control pairs at each PLD, and one calibration image. During these scans, subjects are also instructed to look at a crosshair, blink normally and try not to fall asleep. The original UKB protocol did not include ASL, however this modality has been recently collected on the UKB COVID-19 study participants.

#### 2.2.4 Raw scan quality control

We visually inspected the raw images to identify low quality scans to give an indication of tolerability of the protocol (e.g., motion artifacts that would be indicative of the ability of a memory clinic patient to lay still in the scanner), as well as to identify scans that might be informative about robustness in evaluating the analysis pipeline.

### 2.3 Radiology reports

All clinical scans are reported by an experienced radiologist (PP) in a standardised way, following the guidelines that emerged from a survey of the European Society of Neuroradiology (ESNR)(Vernooij et al., 2019).

Table 2 describes the fields of the structured report, as well as the variables that were derived from it for the patients who consented to the use of clinical data for research.

**Table 2:** Standardised radiology report structure.

Section	Description	Derived variables
Atrophy pattern		
Generalised atrophy	Comment on degree of global brain atrophy	Yes/no, asymmetric (yes/no), global cortical atrophy (none/mild/moderate/severe)
Medial Temporal lobe Atrophy (MTA)	Comment on regional brain atrophy, including MTA scale	Scheltens score left and right MTA
White matter hyperintensities (WMH)	State if present or not. Fazekas score for microangiopathic change.	Fazekas score total WMH, periventricular (PWMH), deep (DWMH), alternative cause (yes/no)
	Is the distribution likely to be microangiopathic change or is there is suspicion of an alternative cause such as multiple sclerosis or CADASIL?	
Microhaemorrhages	State if present or not. If present, comment on likely aetiology based on number and distribution (CAA, hypertension, previous trauma)	Presence (yes/no), count, location
Previous infarction or intracranial haemorrhage	Presence of previous lobar haemorrhage	Presence (yes/no), comments
	Presence of previous lobar or lacunar infarction	
Restricted diffusion suggesting prion disease	Presence of areas of restricted diffusion suggesting prion disease.	Yes/no
Important negatives	Presence of mass, tumours, extra-axial collection, hydrocephalus.	Mass/tumours (yes/no), extra-axial collection (yes/no), hydrocephalus (yes/no)
Other incidental pathologies	Mention specifically any clinically relevant incidental pathology (e.g. developmental brain malformation, sinonasal pathology, skull base pathology, orbital pathology)	Details

CADASIL = Cerebral autosomal dominant arteriopathy with subcortical infarcts and leukoencephalopathy; CAA = Cerebral Amyloid Angiopathy.

### 2.4 BHC MRI analysis: Adapting the UKB pipeline to memory clinic population

The second component of UKB imaging implemented in the BHC is the image analysis pipeline. The pipeline (Alfaro-Almagro et al., 2018) automatically processes the images and extracts imaging derived phenotypes (IDPs), standardised quantitative measures that can be easily used and interpreted also by non-imaging experts. Because of the well-matched acquisition protocol, we were able to apply the pipeline, normally used only in research, to extract quantitative information from clinical scans of BHC patients.

In this work we focused on obtaining the most clinically useful IDPs for dementia diagnosis (i.e. those relevant to the sections included in the standardised radiology report), from amongst the IDPs currently generated by the pipeline (Alfaro-Almagro et al., 2018). We therefore analysed T1-weighted scans and T2-FLAIR scans and extracted measures of global atrophy (total grey matter volume), hippocampal atrophy (hippocampal volume) and white matter change (volume of periventricular and deep WMHs).

We then tested whether the UKB pipeline applied to a real-life memory clinic population was successful in extracting the measures of interest and, where necessary, performed the necessary adjustments to the analysis pipeline.

#### 2.4.1 Image processing with UKB pipeline

Structural images were analysed with FSL using the UKB analysis pipeline (version 1.5 – https://git.fmrib.ox.ac.uk/falmagro/UK_biobank_pipeline_v_1.5)(Alfaro-Almagro et al., 2018). In brief, the main steps of the T1 pipeline that derive the selected IDPs are: gradient distortion correction and defacing, brain extraction, linear and non-linear registration to standard space, FIRST subcortical structure segmentation (Patenaude et al., 2011), FAST bias field correction and tissue-type segmentation (Zhang et al., 2001) along with a shortened version of SIENAX reliant on FAST (Smith et al., 2002). The T2-FLAIR pipeline includes bias field correction, registration to T1 and standard space, brain extraction by masking with the T1 brain mask, and WMH segmentation with BIANCA (Griffanti et al., 2016), further subdivided into periventricular and deep WMH (Griffanti et al., 2018).

#### 2.4.2 UKB pipeline output quality check and optimisation

For the first 32 patients, visual inspection in FSLeyes was performed for the following stages in the UKB structural pipeline: brain extraction of T1 and T2-FLAIR scans, tissue-type segmentation from FAST/SIENAX (with particular attention to grey matter segmentation), hippocampus segmentation output from FIRST and WMH segmentation output from BIANCA. Each stage was rated as high, medium, or low quality by two raters independently (LG, GG), and the final rating was reached through consensus. Notes were included on the type of inaccuracy if present. These quality checks informed selection of the tools requiring optimisation.

**Fig. 1:**
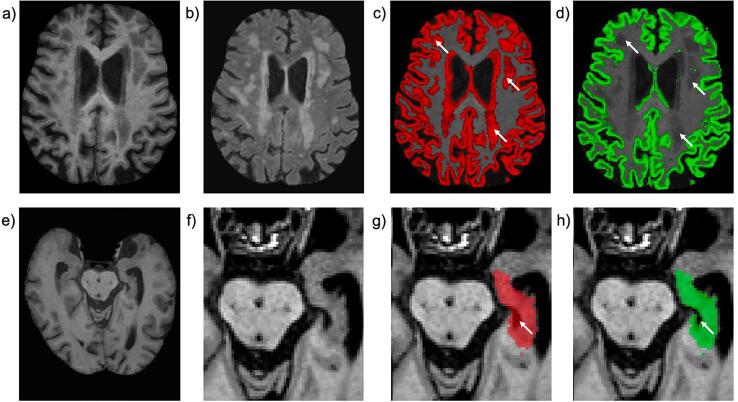
Results of UKB pipeline optimisation for memory clinic population. a) Example T1-weighted scan from a BHC patient where WMHs are T1 hypointense, similar in intensity to grey matter (GM). b) Corresponding T2-FLAIR scan showing WMHs. c) Uncorrected GM segmentation (red), with significant periventricular and deep WMHs classified as GM (white arrows). d) GM segmentation corrected with lesion-masking (green). e) Example T1-weighted scan from a different BHC patient, f) zoomed-in around the hippocampus. g) Uncorrected hippocampus segmentation (red) with errors (inclusion of CSF) highlighted by white arrow. h) CSF-masked hippocampus segmentation removed incorrectly labelled CSF voxels. (For interpretation of the references to colour in this figure legend, the reader is referred to the web version of this article.)

Because this patient population is characterised by increased cortical and medial temporal lobe atrophy and higher white matter hyperintensities load, we identified two analysis steps that required optimisation (Fig. 1).

Grey matter (GM) segmentations (from FAST/SIENAX) were often flagged as inaccurate due to WMHs being misclassified as GM, causing total GM volume to be overestimated. This is due to the fact that WMHs that are bigger and likely at a more severe stage become visible on T1-weighted scans as hypo-intensities (Melazzini et al., 2021). To improve the segmentation accuracy, we implemented in the pipeline a modified version of SIENAX (-lm option). With this option WMHs (segmented with BIANCA) are initially excluded from tissue-type segmentation, then added to the final white matter map (since WMHs are, by definition, part of the WM).

Hippocampal segmentations from FIRST were sometimes flagged as inaccurate in the quality checks. Since by design FIRST does not explicitly avoid the inclusion of non-grey matter tissues within its boundaries, one common error in this population with larger amounts of medial temporal lobe atrophy was the inclusion of cerebrospinal fluid (CSF) regions in the hippocampus. We therefore attempted CSF-masking to improve segmentation accuracy. In brief, this approach takes the CSF partial volume estimate (PVE) map generated by FAST, thresholds it to keep only voxels with high CSF content, and removes CSF areas from the FIRST hippocampal segmentations. The threshold for the CSF PVE map was empirically chosen as the value giving the best trade-off between removing CSF voxels from the hippocampus mask and avoiding under-segmenting the rest of the structure.

Optimised tissue-type and hippocampus segmentations were compared to the initial segmentations in FSLeyes and rated as high, medium, or low quality by the same raters (LG,GG – final rating reached through consensus). The most accurate segmentation strategies (FAST/SIENAX with or without lesion-masking and FIRST with or without CSF masking) were carried forward to subsequent analyses.

#### 2.4.3 Optimised pipeline validation

We performed both an internal and external validation of the optimised analysis pipeline. Prior to any analyses, all IDPs were normalised for head size using the SIENAX scaling factor to correct for this between-subject variability. IDPs that were not normally distributed (Kolmogorov-Smirnov test) were cube-root transformed. The cube-root transformation was chosen as it is a transformation that produces linear units (i.e. mm instead of mm^3^), and which generally produces a transformed variable with a symmetric distribution.

The internal validation was performed by examining the level of agreement between the IDPs extracted from the pipeline with visual ratings from radiology reports. We used ANOVA tests to compare the total GM volume to the global cortical atrophy scale, the left and right hippocampal volumes obtained from FIRST with left and right medial temporal lobe atrophy (MTA) scale (Scheltens et al., 1992), and total WMH, PWMH and DWMH volumes from BIANCA against the Fazekas scale (Fazekas et al., 1987). Where a visual rating score had <5 cases (usually lowest or highest scores), they were grouped with the nearest score for the statistical analyses.

External validation was then performed against non-imaging variables. Based on their known associations with WMHs and atrophy, three clinical variables were used for external validation of tool performance: patient age, the Addenbrooke’s Cognitive Examination-III (ACE-III)(Hsieh et al., 2013) total score and the ACE-III memory sub-score. Among the tests used in memory clinics (Ballard, 2013), the ACE-III was chosen because it provides a comprehensive assessment of five cognitive domains (attention, memory, language, verbal fluency and visuospatial function) and showed higher accuracy for the diagnosis of mild Alzheimer’s disease than other screening tools (Matias-Guiu et al., 2017). Partial correlation (correlation with age controlled for sex, correlation with ACE-III and ACE-III memory score controlled for age and sex) was used to test the relationship between these variables and GM volumes, FIRST hippocampal volumes, and BIANCA WMH volumes, under the hypothesis that strong correlations would indicate high tool performance.

All statistical analyses were performed in SPSS 27 (IBM) and Bonferroni-corrected for multiple comparisons.

### 2.5 Towards quantitative radiology reports for the BHC

Finally, we explored the use of UKB data as a reference population to aid derivation of individual prediction on BHC patients. This would allow incorporating quantitative measures into radiology reports, delivering the enhanced brain information through a decision support tool that is interpretable and useful to clinicians and patients. To this aim, we compared the characteristics of BHC patients with those of the UKB participants, both in terms of demographics and hippocampal volume using previously published nomograms derived from 19,793 generally healthy UKB participants (Nobis et al., 2019).

## 3 Results

### 3.1 Sample characteristics

**Fig. 2:**
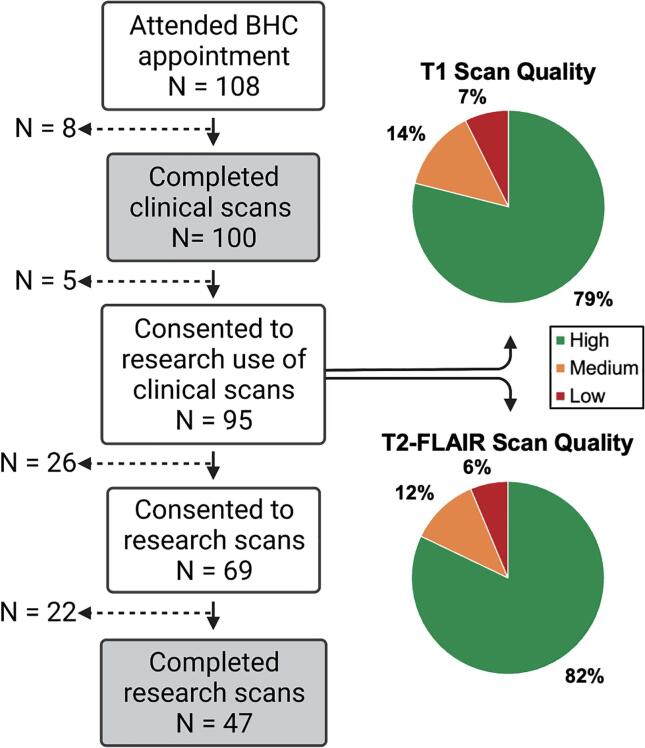
Flow chart of patients attending the Brain Health Clinic (consent and completion for clinical and research scans) and quality assessment of raw structural MRI data for the patients who completed the clinical scans and consented to the use of data for research (N = 95).

From the opening of the BHC in August 2020 to November 2021, 108 patients attended their BHC appointment (O’Donoghue et al., 2022a)). As shown in Fig. 2, 92.6 % (N = 100) completed the clinical scans (2 patients were not scanned due to inability to lie in scanner, 2 had safety contraindications on the day, 1 was claustrophobic, and 3 scans were abandoned due to claustrophobia and discomfort in the scanner). Of these, 95 (88.0 %) patients consented to the use of their clinical MRI data for research and are included here. Sixty-nine patients (63.9 %) consented to undergo additional research scans, and forty-seven (43.5 %) completed all research scans.

Table 3 includes the characteristics of the 95 patients included, a summary of the findings on the radiology reports and the number of scans available for each modality.

**Table 3:** Sample characteristics.

Sample characteristic	
**Demographics (N = 95)**	
Age (years) – mean ± SD (range)	78.4 ± 6.2 (66–101)
Sex (M/F)	47/48

**Cognitive scores (N = 92)**	
ACE-III total score – mean ± SD (range)	73.8 ± 17.1 (9–98)
ACE-III memory sub-score – mean ± SD (range)	15.2 ± 6.5 (0–26)

**Diagnoses (ICD10 code) (N = 95)**	N
Alzheimer’s Disease (F00)	48
Non-Alzheimer’s Dementia (F01, F03)	3
Mild Cognitive Impairment (F06.7)	26
Neuropsychiatric (F31, F32, F41)	7
No Diagnosis	11

**Radiology report (N = 95)**	
Generalised atrophy (counts for none/mild/moderate/severe)	5/57/30/3
L MTA score – median (range) [counts]	2 (0–4) [6/26/32/27/4]
R MTA score – median (range) [counts]	2 (0–4) [6/28/29/29/3]
Fazekas PWMH score – median (range) [counts]	2 (0–3) [3/44/33/15]
Fazekas DWMH score – median (range) [counts]	1 (0–3) [4/54/36/1]
Fazekas total score – median (range) [counts]	3 (0–6) [1/5/38/17/19/14/1]
Microhaemorrhages (yes/no)	10/85
Previous haemorrhages (yes/no)	3/92
Previous chronic or acute infarcts (yes/no)	8/87
Restricted diffusion suggesting prion disease (yes/no)	0/95
Mass (yes/no)	6/89
Extra-axial collection (yes/no)	0/95
Hydrocephalus (yes/no)	0/95

**Clinical sequences (N = 95)**	N
T1	95
T2-FLAIR	95
dMRI 3-scan trace	95
swMRI	95

**Research sequences (N = 51)**	N
dMRI	51
ASL	51
Resting fMRI	47

SD = standard deviation; ACE-III = Addenbrooke’s Cognitive Examination-III; ICD10 = International Classification of Diseases, tenth edition (F00 = Dementia in Alzheimer disease; F01 = Vascular Dementia; F03 = Unspecified dementia; F06.7 = Mild cognitive disorder; F31 = Bipolar affective disorder; F32 = Depressive episode; F41 = Other anxiety disorders); MTA = Medial Temporal lobe Atrophy; PWMH = periventricular white matter hyperintensities; DWMH = deep white matter hyperintensities; FLAIR = Fluid-Attenuated Inversion Recovery; swMRI = susceptibility-weighted imaging; dMRI = diffusion-weighted MRI; fMRI = functional MRI; ASL = Arterial Spin Labeling.

Quality checks of raw T1-weighted and T2-FLAIR images overall show that the UKB MRI sequences were suitable for this clinical population (93 % of T1 scans and 94 % of T2-FLAIR scans were high- or medium-quality, Fig. 2). The most common quality issues were motion artifacts.

### 3.2 Radiology reports

A summary of the main findings from the radiology reports is provided in Table 3.

We observed a level of generalised atrophy that was mostly mild or moderate. Of the three severe cases, two had an atrophy level that was reported as moderate to severe, and one had severe asymmetrical atrophy of the temporal lobe with relative sparing of the rest of the brain. Given this small number of severe cases, in subsequent analyses we merged the moderate and severe global cortical atrophy classes into one category (moderate/severe).

**Fig. 3:**
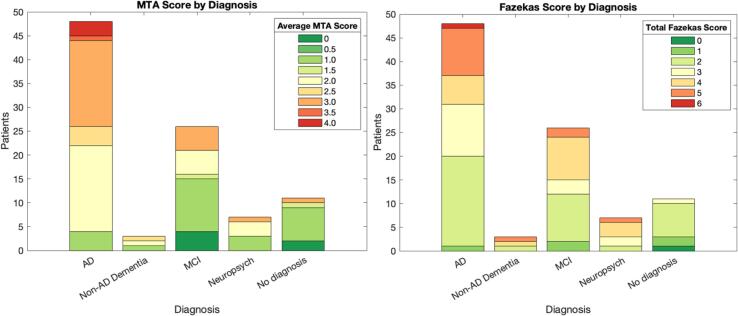
Visual rating scores for hippocampal atrophy (average between left and right MTA score) and white matter hyperintensities (total Fazekas score) for each diagnostic group. MTA = medial temporal lobe atrophy; AD = Alzheimer’s disease; MCI = mild cognitive impairment.

The level of hippocampal atrophy was overall moderate (median MTA = 2). The amount of white matter hyperintensities was predominantly moderate in the periventricular areas and mild in the deep white matter. Fig. 3 summarises the levels of hippocampal atrophy (MTA score) and white matter hyperintensities (Fazekas score) for each diagnostic group.

Three patients had a previous haemorrhage, and 8 patients had a previous infarct (one acute, seven chronic). Of the ten patients presenting microhaemorrhages, in four cases there was a single lesion reported, in three cases there were two lesions, and in the remaining cases there were several lesions (more than five). A mass was reported for six patients: in 3 cases it was a cyst, 2 cases of meningioma, and 1 cavernous haemangioma. No cases of prion disease, extra-axial collection or hydrocephalus were reported.

### 3.3 BHC MRI analysis: Pipeline optimisation and validation

The automated analysis pipeline failed on one participant (unsuccessful T1 brain extraction and registrations, preventing further analyses on other modalities), due to high levels of motion in the T1 scan. The scan was also marked as low-quality image in the raw data QC and the radiology report included a note that images were degraded by movement artifacts (the radiologist was still able to provide a report). For the remaining 94 patients, a useable output was produced, including those for which structural abnormalities were reported (e.g., infarcts or masses). Results are therefore reported for 94 patients.

#### 3.3.1 UKB pipeline output quality check and optimisation

Table 4 shows the results of the visual check on the pipeline outputs on the first 32 patients before and after optimisation. The low number of high-quality tissue-type and hippocampal segmentations with the default pipeline prompted the need for optimising these processing steps. Although several WMH segmentations were rated of medium quality, the most common errors were small false positive clusters in the cortex and overestimation of the lesion size in cases with low WMH load. While there is still room for improvement in WMH segmentation, this was not set out as a priority, as the inaccuracies would not have a big impact on the total volume (and on the lesion masking procedure used to improve tissue-type segmentation). Similarly, the other steps of the pipeline (brain extraction and registration) were deemed of high or medium quality, with small inaccuracies unlikely to significantly affect the calculation of most IDPs.

**Table 4:** UKB pipeline output quality check before and after optimisation on the first 32 BHC patients.

Pipeline output	Before optimisation			After optimisation		
	High	Medium	Low	High	Medium	Low
T1 brain extraction (BET)	25	6	1	–	–	–
Tissue-type segmentation (FAST/SIENAX)	2	10	20	27	5	0
Hippocampus segmentation (FIRST)	13	17	2	18	13	1
T2-FLAIR brain extraction (BET)	25	6	1	–	–	–
White matter hyperintensities (BIANCA)	17	12	3	–	–	–

After implementing the two optimisation strategies, we observed that the quality of the results improved in both cases; lesion masking significantly improved the quality of tissue-type segmentation, while CSF masking only led to a modest improvement of hippocampal segmentation (with a threshold of the CSF PVE map of 0.7 empirically chosen from a range between 0.6 and 0.9). The modifications to the pipeline were deemed successful and volumes extracted with the optimised pipeline were used in further analyses. The presence of other structural abnormalities caused minimal or no error in the segmentations, not influencing the overall quality of the results and estimated IDPs.

#### 3.3.2 Optimised pipeline validation

**Fig. 4:**
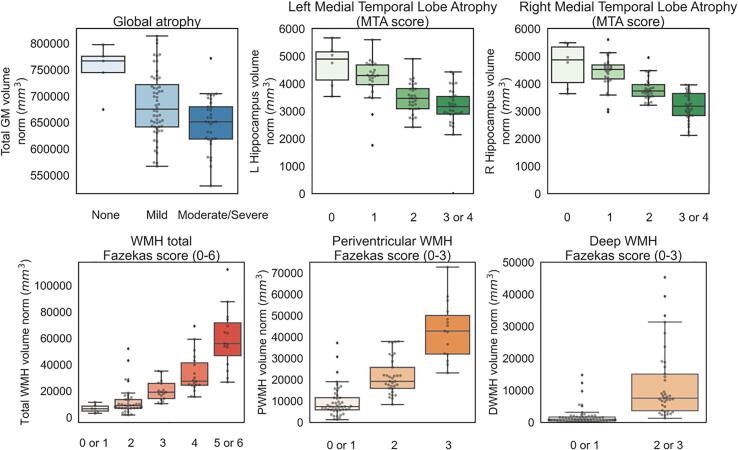
Optimised pipeline internal validation. Values of IDPs (y axes) derived from the automated pipeline, all normalised for head size (WMH volumes were cube-root transformed before entering statistical analyses) against the classifications (x axes) obtained from clinical radiology reports (blind to the results of the pipeline). Scores with<5 cases were grouped with the nearest score for statistical analyses (MTA 3 and 4; Total Fazekas 0 and 1, 5 and 6; PWMH Fazekas 0 and 1; DWMH Fazekas 0 and 1, 2 and 3). See Table 3 for original counts for each score and supplementary Fig. 1 for boxplots with original scores).

Fig. 4 shows the results of the internal validation of the optimised pipeline. Visual rating scores derived from the radiology reports for atrophy and white matter hyperintensities are compared with the corresponding IDPs obtained with the automated pipeline (all normalised for head size, WMH volumes additionally cube-root transformed before entering statistical analysis).

The ANOVA tests showed that there was a statistically significant difference in total GM volume between the different atrophy categories (F(2,91) = 8.682, p < 0.001, partial eta squared = 0.160), a significant difference in hippocampal volumes across MTA scores (Left: F(3,90) = 15.360, p < 0.001, partial eta squared = 0.339; Right: F(3,90) = 32.024, p < 0.001, partial eta squared = 0.516) and a significant difference in white matter hyperintensity volumes across Fazekas scores (Total: F(4,89) = 43.642, p < 0.001, partial eta squared = 0.662; PWMH: F(2,91) = 75.355, p < 0.001, partial eta squared = 0.624; DWMH: H(1,92) = 93.888, p < 0.001, partial eta squared = 0.505). All results survive Bonferroni correction.

Table 5 reports the correlations between the IDPs and age (controlling for sex), total ACE III score and ACE III memory sub-score (controlling for age and sex), performed as an external validation of the optimised pipeline.

**Table 5:** Optimised pipeline external validation.

IDP	mean	st.dev	correlation (p-value)		
			Age	ACE-III total	ACE-III memory
GM total volume	674220.74	61288.37	−0.546 (<0.001)*	0.347 (0.001)*	0.353 (0.001)*
Left hippocampal volume	3619.85	848.84	−0.319 (0.002)*	0.407 (<0.001)*	0.392 (<0.001)*
Right hippocampal volume	3819.95	740.19	−0.464 (<0.001)*	0.081 (0.453)	0.149 (0.164)
Total WMH volume	24845.35	21800.92	0.467 (<0.001)*	−0.383 (<0.001)*	−0.212 (0.047)
PWMH volume	19145.87	14682.35	0.460 (<0.001)*	−0.354 (0.001)*	−0.192 (0.071)
DWMH volume	5699.48	8738.15	0.410 (<0.001)*	−0.331 (0.002)*	−0.207 (0.051)

Mean and standard deviation reported for head size normalised IDPs (all volumes in mm^3^). Due to non-normal distributions, the WMH volumes were cube-root transformed before entering statistical analyses.

Age correlation controlled for sex, ACE-III correlations controlled for age and sex.

IDP = imaging derived phenotype; GM = grey matter; ACE-III = Addenbrooke’s Cognitive Examination-III; WMH = white matter hyperintensities; PWMH = periventricular white matter hyperintensities; DWMH = deep white matter hyperintensities.

* Significant after Bonferroni correction across 18 tests (p < 0.003).

Total GM volume was significantly negatively correlated with age and positively correlated with ACE-III total and memory sub-score. Regarding hippocampal volumes, significant negative correlations were observed between age and both the left and right hippocampi. Left hippocampal volume was also significantly correlated with ACE-III scores (total and memory sub-score), while correlations between right hippocampal volume and ACE-III scores were not significant. We further verified that the association between hippocampal volumes and ACE-III score was significantly different between hemispheres (F(1,89) = 6.062, p = 0.015 on interaction between hemisphere and ACE-III score using a linear mixed model – lmer package in R).

Greater WMH total, periventricular and deep volumes significantly correlated with higher age and lower ACE-III total score. None of the correlations between WMH volumes and ACE-III memory sub-score reached Bonferroni-corrected significance.

### 3.4 Towards quantitative radiology reports for the BHC

**Fig. 5:**
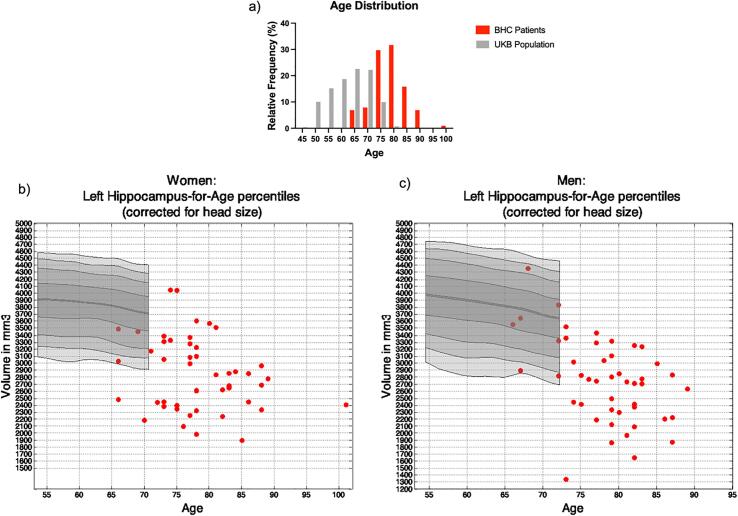
Comparison of BHC and UKB data. a) Histogram of age distribution for 94 BHC patients (red) and 19,793 UKB participants from (Nobis et al., 2019) (grey). b) Left hippocampal volumes of female BHC patients (red) compared with UKB data (grey nomogram); c) Left hippocampal volumes of male BHC patients (red) compared with UKB data (grey nomogram). Most BHC patients fall in the lower percentiles of the nomograms, suggesting potentially pathological deviations from the norm. The remaining BHC patients were outside the nomograms age range, highlighting need for expanded normative data. See supplementary Fig. 2 for right hippocampus results. (For interpretation of the references to colour in this figure legend, the reader is referred to the web version of this article.)

Fig. 5 shows how the BHC patients compare to 19,793 generally healthy UKB participants (Nobis et al., 2019). The age distribution of BHC and UKB participants is significantly different (BHC: 78.4 ± 6.2 years – range 66–101 years; UKB: 63.16 ± 7.50 years – range 45–81 years). Consequently, only a very small minority fall within the age range of the nomograms. For these patients, hippocampal volumes are overall comparable to those from UKB participants, with several patients falling in the lower percentiles.

## 4 Discussion

We have adapted the UK Biobank brain imaging for use in a real-world memory clinic setting, the Oxford Brain Health Clinic. We found that the BHC acquisition protocol was well tolerated by patients. The optimised analysis pipeline produced IDPs of total GM volume, hippocampal volume and WMH volume that were in good agreement with visual ratings from the radiology reports and correlated with factors known to be associated with atrophy and vascular pathology. The age difference between BHC patients and UKB participants highlighted the need for additional scans on elderly healthy controls to improve reference distributions.

By dividing the UKB brain MRI protocol into clinical and research sections we were able to exploit its technical advances to generate high quality data in a reduced time, while tailoring it to inform dementia diagnosis. The high overall quality of raw T1-weighted and T2-FLAIR scans suggests that these UKB-matched sequences are suitable for patients with memory problems. Moreover, by combining clinical scans with optional research add-ons into a single session and enabling patients to opt for their desired level of research participation, this protocol reduces potential barriers to research engagement. Of the 100 patients who were able to complete the clinical scans, 95 consented to the research use of their data. By prioritising data collection with direct clinical benefit and enabling additional research participation, this approach exceeds the target outlined in the UK Prime Minister’s Challenge on Dementia for 10 % of dementia patients to participate in research (UK Department of Health, 2012). This results in a dataset that is representative of the memory clinic population, as the patients are not selectively recruited for a research study. Because patients can also consent to be recontacted for research (current consent rate 73.1 % (O’Donoghue et al., 2022a)), this protocol is also building a valuable participant database for future studies.

There were a number of challenges to adapt the UKB analysis pipeline in this population. Tissue-type segmentation was highly affected by the presence of WMHs, misclassified as GM, due to their similar T1 intensity (Dadar et al., 2018, Melazzini et al., 2021). This inaccuracy has been reported elsewhere with multiple sclerosis (MS) lesions (Battaglini et al., 2012) and WMHs (Dadar et al., 2021). Lesion-filling (i.e. replacing or “filling” the intensity values in the lesion area with intensities that are similar to those in the non-lesion neighbourhood) and lesion-masking (the approach used in this study) are valuable correction tools for MS lesions (Battaglini et al., 2012, Chard et al., 2010). For WMHs of presumed vascular origin, we opted for the use of lesion-masking since WMHs tend to be larger and more confluent than the MS lesions for which lesion-filling was designed (Battaglini et al., 2012). Although alternative methods have been previously described to adjust for WMHs in GM segmentations (Park et al., 2018), lesion-masking provides a straightforward method yielding remarkable improvements in GM segmentation accuracy. Whilst essential in BHC patients with high WMH burdens, lesion-masking was not detrimental in patients with mild WMHs. Overall, the efficacy and simplicity of lesion-masking supports its widespread application in other pipelines and for other populations.

Hippocampal segmentation is notoriously problematic for patients with more atrophy. Previous studies showed better performance of FIRST in non-atrophic brains (Goubran et al., 2020), despite the inclusion of some Alzheimer’s disease patients in its training datasets (Patenaude et al., 2011). Similar inaccuracies have been reported with other hippocampal segmentation tools (Firbank et al., 2008), supporting the widespread need for optimisation. CSF-masking, a novel strategy in this study, led to a modest improvement of the segmentations, despite very small impact on the calculated volumes. This may be because some segmentation inaccuracies also occur at the GM-WM interface, and future efforts should aim to also improve segmentation accuracy at this boundary. Another possibility is that FIRST segmentations may be inaccurate in shape or location but not necessarily in size. In fact, among the segmentations that were still labelled as low quality after CSF-masking, only one was an outlier in terms of volume. Nevertheless, further optimisation is required to improve the quality of these segmentations, especially for future analyses reliant on hippocampal shape (e.g., vertex analysis). Potential alternative approaches include those based on deep learning, like the one proposed by (Dinsdale et al., 2019), which also showed increased performance on Alzheimer’s disease and mild cognitive impairment (MCI) scans after transfer learning (Balboni et al., 2022) or the one by (Liu et al., 2020), which incorporates hippocampal segmentation and Alzheimer’s disease classification. It will be important to test these and other new approaches that might be included in future versions of the UKB pipeline in an unselected clinical population like the BHC. This demonstrates the value of sharing BHC data for the development of these methods.

From our internal validation we found good agreement between IDPs and visual ratings, suggesting that the pipeline can be used to automatically extract meaningful information for memory clinic patients. Our external validation strategy confirmed the well-known associations between age and both atrophy and vascular pathology. We also found that white matter hyperintensity volume was associated with impaired cognitive function, which is consistent with previous studies (Bolandzadeh et al., 2012, Griffanti et al., 2018, Kim et al., 2008). ACE-III scores (total and memory) were significantly associated with the left hippocampal volume, but not the right, with a significant interaction between hemisphere and ACE-III score in the linear mixed model. The left hippocampus has been previously found to be smaller and more strongly correlated with cognitive symptoms in MCI and dementia than the right hippocampus (Ezzati et al., 2016, Muller et al., 2005, Shi et al., 2009). Compared to the right hippocampus, we see smaller left hippocampal volumes with greater variability both before and after correction with CSF-masking (Table 5, paired *t*-test L < R hippocampus p = 0.003). This smaller variability may also limit our power to detect correlations with ACE-III total and memory scores on the right side.

Finally, we explored the alignment between the BHC and UKB populations using previously published nomograms for hippocampal volume (Nobis et al., 2019). There is a substantial age difference between UKB participants and BHC patients, meaning that UKB data are currently not suitable to be used as reference population for memory clinic patients. Nomograms used here were generated using 19,793 participants, although data from the latest release including 51,532 participants who underwent imaging (visit 2 – see supplementary Fig. 3) are very similar, with a mean age of 64.54 ± 7.81 and age range between 44 and 83 years. The use of a sliding window approach further restricts the age range, since the nomograms were designed so that each window contained 10 % of the participants (Nobis et al., 2019). Using alternative fitting methods can help building nomograms that cover a wider age range (Fraza et al., 2021), provided that the uncertainty, especially when fewer samples are available (e.g., either side of the age range), is carefully considered (Bozek et al., 2022). Recently, ‘brain charts’ of the four main tissue volumes of the cerebrum (total cortical GM volume, total WM volume, total subcortical GM volume and total ventricular CSF volume) have been generated from over 100,000 scans (including UKB) across the lifespan (Bethlehem et al., 2022). They include the age range of BHC patients, but being generated from multi-site data, they currently require at least 100 scans from healthy controls to estimate study-specific offset. Moreover, these charts are not yet extended to more fine-grained IDPs that are likely to be more useful for dementia diagnosis, but also more sensitive in variations in image quality. Despite the current open challenges, the very active field of normative modelling research has the potential to ultimately enable the integration of IDPs and reference distributions in an imaging decision support tool for dementia diagnosis, the clinical utility of which can be directly assessed in the clinical setting at the BHC.

There are a number of methodological considerations when interpreting these results. Firstly, although the BHC population is non-selective and highly representative of the local memory clinic population, the local population of South Oxfordshire lacks the ethnic diversity of other areas of the country. We hope that by sharing our protocol, analysis pipeline and data as well as collaborating with other brain health clinic initiatives we will contribute to building a more representative dataset of memory clinic patients. Secondly, as described above, the UK Biobank is an impressively large, but nevertheless incomplete reference sample because the age range does not extend to cover that of the memory clinic population. The repeat imaging within the UK Biobank will go some way to address this, and we are currently acquiring additional scans on elderly healthy controls with the same protocol to improve reference distributions. Finally, while the analyses described here give confidence in the use of the UK Biobank acquisition and analysis protocols for the memory clinic population as a whole, we do not yet have an appropriate set of controls and sufficient numbers of patients in each distinct diagnostic group to perform group comparisons. The UKB pipeline produces thousands of IDPs and a sample size calculation will always depend on the specific research question. As such, we have not generated power calculations for individual comparisons. There is a literature on sample size calculations for hippocampal size (i.e. a single IDP), where power calculations and ROC curve analysis have been investigated formally. Very similar classification performance (controls vs Alzheimer’s disease AUC = 0.85–0.9; controls vs MCI AUC = 0.7–0.8) has been obtained with a highly variable number of subjects (from<50 in each group to hundreds) (Colliot et al., 2008, Estevez-Sante et al., 2020, Jimenez-Huete et al., 2017, Landau et al., 2010, Voevodskaya et al., 2014), often from the same dataset (ADNI) (Estevez-Sante et al., 2020, Jimenez-Huete et al., 2017, Landau et al., 2010, Voevodskaya et al., 2014). The scope of this paper was to present the BHC acquisition protocol, the adapted analysis pipeline and the data, that we hope will contribute to a number of future studies.

To conclude, to the best of our knowledge, this is the first time that UKB imaging (acquisition, analysis pipeline and reference data) has been adapted and applied for an unselected real-world patient population. The UKB pipeline is regularly updated to include new contributions and technical advances in image processing. This could include the automatic detection and quantification of other brain characteristics that are currently assessed in the clinical setting for dementia (e.g., other signs of small vessel disease like lacunes or microhaemorrhages, other structural abnormalities like infarcts, masses or haemorrhages). Having access to data from a real-world memory clinic population helps inform these methodological developments at the same time as testing their clinical utility individually and in combination with other imaging and non-imaging variables (e.g. lifestyle factors, health measures and genetics), thus bridging the gap between research and clinical practice.

## Data/Code availability statement

The complete BHC MRI protocol and scanning procedure is available through the WIN MR Protocols Database at: https://open.win.ox.ac.uk/protocols/stable/6974395a-3745–4861-b8cc-1887e787d1c4 (O’Donoghue et al., 2022b).

The UK Biobank brain MRI analysis pipeline used in this study (v1.5) is openly available (https://git.fmrib.ox.ac.uk/falmagro/uk_biobank_pipeline_v_1.5/-/tree/master). Modified or additional scripts for the analyses performed in this study are available at (https://git.fmrib.ox.ac.uk/open-science/analysis/brain-health-clinic-mri).

The MRI data presented in this paper will be available via the Dementias Platform UK (https://portal.dementiasplatform.uk/CohortDirectory/Item?fingerPrintID=BHC) and access will be granted through an application process, reviewed by the BHC Data Access Group. The BHC Data Access Group will start accepting applications to access BHC data upon publication of the present work. Data will continue to be released in batches as the BHC progresses in order to minimise the risk of participant identification.

The MRI acquisition protocol and the analysis pipeline code are openly available. The MRI data will be available via the Dementias Platform UK (details in supplementary material).

## CRediT authorship contribution statement

**Ludovica Griffanti:** Conceptualization, Methodology, Software, Investigation, Data curation, Formal analysis, Validation, Visualization, Writing – original draft. **Grace Gillis:** Methodology, Software, Investigation, Data curation, Formal analysis, Validation, Visualization, Writing – original draft. **M. Clare O'Donoghue:** Conceptualization, Methodology, Investigation, Data curation, Project administration, Writing – review & editing. **Jasmine Blane:** Investigation, Data curation, Project administration, Writing – review & editing. **Pieter M. Pretorius:** Conceptualization, Investigation, Writing – review & editing. **Robert Mitchell:** Investigation, Writing – review & editing. **Nicola Aikin:** Investigation, Resources, Writing – review & editing. **Karen Lindsay:** Data curation, Project administration, Writing – review & editing. **Jon Campbell:** Investigation, Resources, Writing – review & editing. **Juliet Semple:** Investigation, Resources, Writing – review & editing. **Fidel Alfaro-Almagro:** Methodology, Software, Writing – review & editing. **Stephen M. Smith:** Methodology, Software, Funding acquisition, Writing – review & editing. **Karla L. Miller:** Methodology, Software, Funding acquisition, Writing – review & editing. **Lola Martos:** Conceptualization, Resources, Funding acquisition, Writing – review & editing. **Vanessa Raymont:** Conceptualization, Resources, Funding acquisition, Supervision, Writing – review & editing. **Clare E. Mackay:** Conceptualization, Methodology, Resources, Funding acquisition, Supervision, Writing – review & editing.

## Declaration of Competing Interest

CEM is a co-founder and shareholder of Exprodo Software, which was used to develop the BHC database. CEM serves on a Biogen Brain Health Consortium (unpaid). No other competing interests to report.

## References

[b0005] Alfaro-Almagro F., Jenkinson M., Bangerter N.K., Andersson J.L.R., Griffanti L., Douaud G., Sotiropoulos S.N., Jbabdi S., Hernandez-Fernandez M., Vallee E., Vidaurre D., Webster M., McCarthy P., Rorden C., Daducci A., Alexander D.C., Zhang H., Dragonu I., Matthews P.M., Miller K.L., Smith S.M. (2018). Image processing and Quality Control for the first 10,000 brain imaging datasets from UK Biobank. Neuroimage.

[b0010] Balboni E., Nocetti L., Carbone C., Dinsdale N., Genovese M., Guidi G., Malagoli M., Chiari A., Namburete A.I.L., Jenkinson M., Zamboni G. (2022). The impact of transfer learning on 3D deep learning convolutional neural network segmentation of the hippocampus in mild cognitive impairment and Alzheimer disease subjects. Hum Brain Mapp.

[b0015] Ballard, C.B., A.; Corbett, A.; Livingston, G.; Rasmussen, J., 2013. Helping you to assess cognition. A practical toolkit for clinicians.

[b0020] Battaglini M., Jenkinson M., De Stefano N. (2012). Evaluating and reducing the impact of white matter lesions on brain volume measurements. Hum Brain Mapp.

[b0025] Bethlehem, R.A.I., Seidlitz, J., White, S.R., Vogel, J.W., Anderson, K.M., Adamson, C., Adler, S., Alexopoulos, G.S., Anagnostou, E., Areces-Gonzalez, A., Astle, D.E., Auyeung, B., Ayub, M., Bae, J., Ball, G., Baron-Cohen, S., Beare, R., Bedford, S.A., Benegal, V., Beyer, F., Blangero, J., Blesa Cabez, M., Boardman, J.P., Borzage, M., Bosch-Bayard, J.F., Bourke, N., Calhoun, V.D., Chakravarty, M.M., Chen, C., Chertavian, C., Chetelat, G., Chong, Y.S., Cole, J.H., Corvin, A., Costantino, M., Courchesne, E., Crivello, F., Cropley, V.L., Crosbie, J., Crossley, N., Delarue, M., Delorme, R., Desrivieres, S., Devenyi, G.A., Di Biase, M.A., Dolan, R., Donald, K.A., Donohoe, G., Dunlop, K., Edwards, A.D., Elison, J.T., Ellis, C.T., Elman, J.A., Eyler, L., Fair, D.A., Feczko, E., Fletcher, P.C., Fonagy, P., Franz, C.E., Galan-Garcia, L., Gholipour, A., Giedd, J., Gilmore, J.H., Glahn, D.C., Goodyer, I.M., Grant, P.E., Groenewold, N.A., Gunning, F.M., Gur, R.E., Gur, R.C., Hammill, C.F., Hansson, O., Hedden, T., Heinz, A., Henson, R.N., Heuer, K., Hoare, J., Holla, B., Holmes, A.J., Holt, R., Huang, H., Im, K., Ipser, J., Jack, C.R., Jr., Jackowski, A.P., Jia, T., Johnson, K.A., Jones, P.B., Jones, D.T., Kahn, R.S., Karlsson, H., Karlsson, L., Kawashima, R., Kelley, E.A., Kern, S., Kim, K.W., Kitzbichler, M.G., Kremen, W.S., Lalonde, F., Landeau, B., Lee, S., Lerch, J., Lewis, J.D., Li, J., Liao, W., Liston, C., Lombardo, M.V., Lv, J., Lynch, C., Mallard, T.T., Marcelis, M., Markello, R.D., Mathias, S.R., Mazoyer, B., McGuire, P., Meaney, M.J., Mechelli, A., Medic, N., Misic, B., Morgan, S.E., Mothersill, D., Nigg, J., Ong, M.Q.W., Ortinau, C., Ossenkoppele, R., Ouyang, M., Palaniyappan, L., Paly, L., Pan, P.M., Pantelis, C., Park, M.M., Paus, T., Pausova, Z., Paz-Linares, D., Pichet Binette, A., Pierce, K., Qian, X., Qiu, J., Qiu, A., Raznahan, A., Rittman, T., Rodrigue, A., Rollins, C.K., Romero-Garcia, R., Ronan, L., Rosenberg, M.D., Rowitch, D.H., Salum, G.A., Satterthwaite, T.D., Schaare, H.L., Schachar, R.J., Schultz, A.P., Schumann, G., Scholl, M., Sharp, D., Shinohara, R.T., Skoog, I., Smyser, C.D., Sperling, R.A., Stein, D.J., Stolicyn, A., Suckling, J., Sullivan, G., Taki, Y., Thyreau, B., Toro, R., Traut, N., Tsvetanov, K.A., Turk-Browne, N.B., Tuulari, J.J., Tzourio, C., Vachon-Presseau, E., Valdes-Sosa, M.J., Valdes-Sosa, P.A., Valk, S.L., van Amelsvoort, T., Vandekar, S.N., Vasung, L., Victoria, L.W., Villeneuve, S., Villringer, A., Vertes, P.E., Wagstyl, K., Wang, Y.S., Warfield, S.K., Warrier, V., Westman, E., Westwater, M.L., Whalley, H.C., Witte, A.V., Yang, N., Yeo, B., Yun, H., Zalesky, A., Zar, H.J., Zettergren, A., Zhou, J.H., Ziauddeen, H., Zugman, A., Zuo, X.N., R, B., Aibl, Alzheimer's Disease Neuroimaging, I., Alzheimer's Disease Repository Without Borders, I., Team, C., Cam, C.A.N., Ccnp, Cobre, cVeda, Group, E.D.B.A.W., Developing Human Connectome, P., FinnBrain, Harvard Aging Brain, S., Imagen, Kne, Mayo Clinic Study of, A., Nspn, Pond, Group, P.-A.R., Vetsa, Bullmore, E.T., Alexander-Bloch, A.F., 2022. Brain charts for the human lifespan. Nature 604, 525-533.

[b0030] Biondo F., Jewell A., Pritchard M., Aarsland D., Steves C.J., Mueller C., Cole J.H. (2022). Brain-age is associated with progression to dementia in memory clinic patients. Neuroimage Clin.

[b0035] Bolandzadeh N., Davis J.C., Tam R., Handy T.C., Liu-Ambrose T. (2012). The association between cognitive function and white matter lesion location in older adults: a systematic review. BMC Neurol.

[b0040] Bosco P., Redolfi A., Bocchetta M., Ferrari C., Mega A., Galluzzi S., Austin M., Chincarini A., Collins D.L., Duchesne S., Marechal B., Roche A., Sensi F., Wolz R., Alegret M., Assal F., Balasa M., Bastin C., Bougea A., Emek-Savas D.D., Engelborghs S., Grimmer T., Grosu G., Kramberger M.G., Lawlor B., Mandic Stojmenovic G., Marinescu M., Mecocci P., Molinuevo J.L., Morais R., Niemantsverdriet E., Nobili F., Ntovas K., O'Dwyer S., Paraskevas G.P., Pelini L., Picco A., Salmon E., Santana I., Sotolongo-Grau O., Spiru L., Stefanova E., Popovic K.S., Tsolaki M., Yener G.G., Zekry D., Frisoni G.B. (2017). The impact of automated hippocampal volumetry on diagnostic confidence in patients with suspected Alzheimer's disease: A European Alzheimer's Disease Consortium study. Alzheimers Dement.

[b0045] Bozek, J., Griffanti, L., Lau, S., Jenkinson, M., 2022. Normative models for neuroimaging markers: Impact of model selection, sample size and evaluation criteria. bioRxiv, 2022.2009.2022.509002.10.1016/j.neuroimage.2023.11986436621581

[b0050] Chard D.T., Jackson J.S., Miller D.H., Wheeler-Kingshott C.A. (2010). Reducing the impact of white matter lesions on automated measures of brain gray and white matter volumes. J Magn Reson Imaging.

[b0055] Colliot O., Chetelat G., Chupin M., Desgranges B., Magnin B., Benali H., Dubois B., Garnero L., Eustache F., Lehericy S. (2008). Discrimination between Alzheimer disease, mild cognitive impairment, and normal aging by using automated segmentation of the hippocampus. Radiology.

[b0060] Dadar, M., Maranzano, J., Ducharme, S., Carmichael, O.T., Decarli, C., Collins, D.L., Alzheimer's Disease Neuroimaging, I., 2018. Validation of T1w-based segmentations of white matter hyperintensity volumes in large-scale datasets of aging. Hum Brain Mapp 39, 1093-1107. 10.1002/hbm.23894 PMC6866430 29181872

[b0065] Dadar, M., Potvin, O., Camicioli, R., Duchesne, S., Alzheimer's Disease Neuroimaging, I., 2021. Beware of white matter hyperintensities causing systematic errors in FreeSurfer gray matter segmentations! Hum Brain Mapp 42, 2734-2745. 10.1002/hbm.25398 PMC8127151 33783933

[b0070] de la Torre J.C. (2012). Cardiovascular risk factors promote brain hypoperfusion leading to cognitive decline and dementia. Cardiovasc Psychiatry Neurol.

[b0075] Dickie D.A., Valdes Hernandez M.D.C., Makin S.D., Staals J., Wiseman S.J., Bastin M.E., Wardlaw J.M. (2018). The brain health index: Towards a combined measure of neurovascular and neurodegenerative structural brain injury. Int J Stroke.

[b0080] Dinsdale N.K., Jenkinson M., Namburete A.I.L. (2019).

[b0085] Estevez-Sante, S., Jimenez-Huete, A., group, A., 2020. Comparative analysis of methods of volume adjustment in hippocampal volumetry for the diagnosis of Alzheimer disease. J Neuroradiol 47, 161-165. 10.1016/j.neurad.2019.02.004 30857897

[b0090] Ezzati A., Katz M.J., Zammit A.R., Lipton M.L., Zimmerman M.E., Sliwinski M.J., Lipton R.B. (2016). Differential association of left and right hippocampal volumes with verbal episodic and spatial memory in older adults. Neuropsychologia.

[b0095] Fazekas F., Chawluk J.B., Alavi A., Hurtig H.I., Zimmerman R.A. (1987). MR signal abnormalities at 1.5 T in Alzheimer's dementia and normal aging. AJR Am J Roentgenol.

[b0100] Filippi, M., Agosta, F., Barkhof, F., Dubois, B., Fox, N.C., Frisoni, G.B., Jack, C.R., Johannsen, P., Miller, B.L., Nestor, P.J., Scheltens, P., Sorbi, S., Teipel, S., Thompson, P.M., Wahlund, L.O., European Federation of the Neurologic, S., 2012. EFNS task force: the use of neuroimaging in the diagnosis of dementia. Eur J Neurol 19, e131-140, 1487-1501. 10.1111/j.1468-1331.2012.03859.x 22900895

[b0105] Firbank M.J., Barber R., Burton E.J., O'Brien J.T. (2008). Validation of a fully automated hippocampal segmentation method on patients with dementia. Hum Brain Mapp.

[b0110] Fraza C.J., Dinga R., Beckmann C.F., Marquand A.F. (2021). Warped Bayesian linear regression for normative modelling of big data. Neuroimage.

[b0115] Frisoni G.B., Fox N.C., Jack C.R., Scheltens P., Thompson P.M. (2010). The clinical use of structural MRI in Alzheimer disease. Nat Rev Neurol.

[b0120] Goodkin O., Pemberton H., Vos S.B., Prados F., Sudre C.H., Moggridge J., Cardoso M.J., Ourselin S., Bisdas S., White M., Yousry T., Thornton J., Barkhof F. (2019). The quantitative neuroradiology initiative framework: application to dementia. Br J Radiol.

[b0125] Gorelick, P.B., Scuteri, A., Black, S.E., Decarli, C., Greenberg, S.M., Iadecola, C., Launer, L.J., Laurent, S., Lopez, O.L., Nyenhuis, D., Petersen, R.C., Schneider, J.A., Tzourio, C., Arnett, D.K., Bennett, D.A., Chui, H.C., Higashida, R.T., Lindquist, R., Nilsson, P.M., Roman, G.C., Sellke, F.W., Seshadri, S., American Heart Association Stroke Council, C.o.E., Prevention, C.o.C.N.C.o.C.R., Intervention, Council on Cardiovascular, S., Anesthesia, 2011. Vascular contributions to cognitive impairment and dementia: a statement for healthcare professionals from the american heart association/american stroke association. Stroke 42, 2672-2713. 10.1161/STR.0b013e3182299496 PMC3778669 21778438

[b0130] Goubran M., Ntiri E.E., Akhavein H., Holmes M., Nestor S., Ramirez J., Adamo S., Ozzoude M., Scott C., Gao F., Martel A., Swardfager W., Masellis M., Swartz R., MacIntosh B., Black S.E. (2020). Hippocampal segmentation for brains with extensive atrophy using three-dimensional convolutional neural networks. Hum Brain Mapp.

[b0135] Griffanti L., Zamboni G., Khan A., Li L., Bonifacio G., Sundaresan V., Schulz U.G., Kuker W., Battaglini M., Rothwell P.M., Jenkinson M. (2016). BIANCA (Brain Intensity AbNormality Classification Algorithm): A new tool for automated segmentation of white matter hyperintensities. Neuroimage.

[b0140] Griffanti L., Jenkinson M., Suri S., Zsoldos E., Mahmood A., Filippini N., Sexton C.E., Topiwala A., Allan C., Kivimaki M., Singh-Manoux A., Ebmeier K.P., Mackay C.E., Zamboni G. (2018). Classification and characterization of periventricular and deep white matter hyperintensities on MRI: A study in older adults. Neuroimage.

[b0145] Hsieh S., Schubert S., Hoon C., Mioshi E., Hodges J.R. (2013). Validation of the Addenbrooke's Cognitive Examination III in frontotemporal dementia and Alzheimer's disease. Dement Geriatr Cogn Disord.

[b0150] Iturria-Medina, Y., Sotero, R.C., Toussaint, P.J., Mateos-Perez, J.M., Evans, A.C., Alzheimer's Disease Neuroimaging, I., 2016. Early role of vascular dysregulation on late-onset Alzheimer's disease based on multifactorial data-driven analysis. Nat Commun 7, 11934. 10.1038/ncomms11934 PMC4919512 27327500

[b0155] Jimenez-Huete, A., Estevez-Sante, S., group, A., 2017. The anteroposterior and primary-to-posterior limbic ratios as MRI-derived volumetric markers of Alzheimer's disease. J Neurol Sci 378, 110-119. 10.1016/j.jns.2017.04.046 PMC5535801 28566144

[b0160] Kim K.W., MacFall J.R., Payne M.E. (2008). Classification of white matter lesions on magnetic resonance imaging in elderly persons. Biol Psychiatry.

[b0165] Landau, S.M., Harvey, D., Madison, C.M., Reiman, E.M., Foster, N.L., Aisen, P.S., Petersen, R.C., Shaw, L.M., Trojanowski, J.Q., Jack, C.R., Jr., Weiner, M.W., Jagust, W.J., Alzheimer's Disease Neuroimaging, I., 2010. Comparing predictors of conversion and decline in mild cognitive impairment. Neurology 75, 230-238. 10.1212/WNL.0b013e3181e8e8b8 PMC2906178 20592257

[b0170] Littlejohns T.J., Holliday J., Gibson L.M., Garratt S., Oesingmann N., Alfaro-Almagro F., Bell J.D., Boultwood C., Collins R., Conroy M.C., Crabtree N., Doherty N., Frangi A.F., Harvey N.C., Leeson P., Miller K.L., Neubauer S., Petersen S.E., Sellors J., Sheard S., Smith S.M., Sudlow C.L.M., Matthews P.M., Allen N.E. (2020). The UK Biobank imaging enhancement of 100,000 participants: rationale, data collection, management and future directions. Nat Commun.

[b0175] Liu, M., Li, F., Yan, H., Wang, K., Ma, Y., Alzheimer's Disease Neuroimaging, I., Shen, L., Xu, M., 2020. A multi-model deep convolutional neural network for automatic hippocampus segmentation and classification in Alzheimer's disease. Neuroimage 208, 116459. 10.1016/j.neuroimage.2019.116459 31837471

[b0180] Matias-Guiu J.A., Valles-Salgado M., Rognoni T., Hamre-Gil F., Moreno-Ramos T., Matias-Guiu J. (2017). Comparative Diagnostic Accuracy of the ACE-III, MIS, MMSE, MoCA, and RUDAS for Screening of Alzheimer Disease. Dement Geriatr Cogn Disord.

[b0185] McKhann G.M., Knopman D.S., Chertkow H., Hyman B.T., Jack C.R., Kawas C.H., Klunk W.E., Koroshetz W.J., Manly J.J., Mayeux R., Mohs R.C., Morris J.C., Rossor M.N., Scheltens P., Carrillo M.C., Thies B., Weintraub S., Phelps C.H. (2011). The diagnosis of dementia due to Alzheimer's disease: recommendations from the National Institute on Aging-Alzheimer's Association workgroups on diagnostic guidelines for Alzheimer's disease. Alzheimers Dement.

[b0190] Melazzini L., Mackay C.E., Bordin V., Suri S., Zsoldos E., Filippini N., Mahmood A., Sundaresan V., Codari M., Duff E., Singh-Manoux A., Kivimaki M., Ebmeier K.P., Jenkinson M., Sardanelli F., Griffanti L. (2021). White matter hyperintensities classified according to intensity and spatial location reveal specific associations with cognitive performance. Neuroimage Clin.

[b0195] Miller K.L., Alfaro-Almagro F., Bangerter N.K., Thomas D.L., Yacoub E., Xu J., Bartsch A.J., Jbabdi S., Sotiropoulos S.N., Andersson J.L., Griffanti L., Douaud G., Okell T.W., Weale P., Dragonu I., Garratt S., Hudson S., Collins R., Jenkinson M., Matthews P.M., Smith S.M. (2016). Multimodal population brain imaging in the UK Biobank prospective epidemiological study. Nat Neurosci.

[b0200] Muller M.J., Greverus D., Dellani P.R., Weibrich C., Wille P.R., Scheurich A., Stoeter P., Fellgiebel A. (2005). Functional implications of hippocampal volume and diffusivity in mild cognitive impairment. Neuroimage.

[b0205] Nobis L., Manohar S.G., Smith S.M., Alfaro-Almagro F., Jenkinson M., Mackay C.E., Husain M. (2019). Hippocampal volume across age: Nomograms derived from over 19,700 people in UK Biobank. Neuroimage Clin.

[b0210] O’Donoghue, M.C., Blane, J., Gillis, G., Mitchell, R., Lindsay, K., Semple, J., Pretorius, P.M., Griffanti, L., Fossey, J., Raymont, V., Martos, L., Mackay, C.E., 2022a. The Oxford Brain Health Clinic: Protocol and Research Database. medRxiv. 10.1136/bmjopen-2022-067808 PMC10407419 37541753

[b0215] O’Donoghue, M.C., Blane, J., Semple, J., Rieger, S., Aikin, N., Campbell, J., Pretorius, P., Griffanti, L., Gillis, G., Okell, T.W., Chiew, M., Smith, S.M., Miller, K.L., Mackay, C.E., 2022b. WIN MR Protocol: Oxford Brain Health Centre (2019_102_BHC). Zenodo.

[b0220] Park B.Y., Lee M.J., Lee S.H., Cha J., Chung C.S., Kim S.T., Park H. (2018). DEWS (DEep White matter hyperintensity Segmentation framework): A fully automated pipeline for detecting small deep white matter hyperintensities in migraineurs. Neuroimage Clin.

[b0225] Patenaude B., Smith S.M., Kennedy D.N., Jenkinson M. (2011). A Bayesian model of shape and appearance for subcortical brain segmentation. Neuroimage.

[b0230] Scheltens P., Leys D., Barkhof F., Huglo D., Weinstein H.C., Vermersch P., Kuiper M., Steinling M., Wolters E.C., Valk J. (1992). Atrophy of medial temporal lobes on MRI in “probable” Alzheimer's disease and normal ageing: diagnostic value and neuropsychological correlates. J Neurol Neurosurg Psychiatry.

[b0235] Scheltens P., Fox N., Barkhof F., De Carli C. (2002). Structural magnetic resonance imaging in the practical assessment of dementia: beyond exclusion. Lancet Neurol.

[b0240] Shi F., Liu B., Zhou Y., Yu C., Jiang T. (2009). Hippocampal volume and asymmetry in mild cognitive impairment and Alzheimer's disease: Meta-analyses of MRI studies. Hippocampus.

[b0245] Smith E.E., Biessels G.J., De Guio F., de Leeuw F.E., Duchesne S., During M., Frayne R., Ikram M.A., Jouvent E., MacIntosh B.J., Thrippleton M.J., Vernooij M.W., Adams H., Backes W.H., Ballerini L., Black S.E., Chen C., Corriveau R., DeCarli C., Greenberg S.M., Gurol M.E., Ingrisch M., Job D., Lam B.Y.K., Launer L.J., Linn J., McCreary C.R., Mok V.C.T., Pantoni L., Pike G.B., Ramirez J., Reijmer Y.D., Romero J.R., Ropele S., Rost N.S., Sachdev P.S., Scott C.J.M., Seshadri S., Sharma M., Sourbron S., Steketee R.M.E., Swartz R.H., van Oostenbrugge R., van Osch M., van Rooden S., Viswanathan A., Werring D., Dichgans M., Wardlaw J.M. (2019). Harmonizing brain magnetic resonance imaging methods for vascular contributions to neurodegeneration. Alzheimers Dement (Amst).

[b0250] Smith S.M., Zhang Y., Jenkinson M., Chen J., Matthews P.M., Federico A., De Stefano N. (2002). Accurate, robust, and automated longitudinal and cross-sectional brain change analysis. Neuroimage.

[b0255] Smith, S.M.A.-A., F.; Miller, K. L., 2020. UK Biobank Brain Imaging Documentation – version 1.8.

[b0260] Staffaroni A.M., Elahi F.M., McDermott D., Marton K., Karageorgiou E., Sacco S., Paoletti M., Caverzasi E., Hess C.P., Rosen H.J., Geschwind M.D. (2017). Neuroimaging in Dementia. Semin Neurol.

[b0265] UK Department of Health, O.p.a.d.t., 2012. Prime Minister’s Challenge on Dementia.

[b0270] van Straaten E.C., Scheltens P., Barkhof F. (2004). MRI and CT in the diagnosis of vascular dementia. J Neurol Sci.

[b0275] Vernooij M.W., Jasperse B., Steketee R., Koek M., Vrooman H., Ikram M.A., Papma J., van der Lugt A., Smits M., Niessen W.J. (2018). Automatic normative quantification of brain tissue volume to support the diagnosis of dementia: A clinical evaluation of diagnostic accuracy. Neuroimage Clin.

[b0280] Vernooij M.W., Pizzini F.B., Schmidt R., Smits M., Yousry T.A., Bargallo N., Frisoni G.B., Haller S., Barkhof F. (2019). Dementia imaging in clinical practice: a European-wide survey of 193 centres and conclusions by the ESNR working group. Neuroradiology.

[b0285] Voevodskaya, O., Simmons, A., Nordenskjold, R., Kullberg, J., Ahlstrom, H., Lind, L., Wahlund, L.O., Larsson, E.M., Westman, E., Alzheimer's Disease Neuroimaging, I., 2014. The effects of intracranial volume adjustment approaches on multiple regional MRI volumes in healthy aging and Alzheimer's disease. Front Aging Neurosci 6, 264. 10.3389/fnagi.2014.00264 PMC4188138 25339897

[b0290] Zhang Y., Brady M., Smith S. (2001). Segmentation of brain MR images through a hidden Markov random field model and the expectation-maximization algorithm. IEEE Trans Med Imaging.

